# Alleleauto: a pipeline for allele identification and analysis of allele-specific gene expression with haplotype-resolved diploid genome assemblies

**DOI:** 10.1016/j.abiote.2026.100056

**Published:** 2026-05-19

**Authors:** Tian-Le Shi, Shuai Nie, Yu-Tao Bao, Zhi-Chao Li, Zhao-Yang Chen, Shi-Wei Zhao, Xue-Mei Yan, Hai-Yao Ma, Xue-Chan Tian, Kai-Hua Jia, Jing-Fang Guo, Jun-Ke Zhang, Jian-Feng Mao

**Affiliations:** a Key Laboratory of Biology and Genetic Improvement of Horticultural Crops (North China), Institute of Forestry and Pomology, Beijing Academy of Agriculture and Forestry Sciences, Beijing, 100093, China; b Rice Research Institute, Guangdong Academy of Agricultural Sciences, Guangdong Key Laboratory of Rice Science and Technology; Guangdong Rice Engineering Laboratory; Key Laboratory of Genetics and Breeding of High Quality Rice in Southern China (Co-construction by Ministry and Province), Ministry of Agriculture and Rural Affairs, Guangzhou, 510640, China; c State Key Laboratory of Tree Genetics and Breeding, National Engineering Research Center of Tree Breeding and Ecological Restoration, National Engineering Laboratory for Tree Breeding, Key Laboratory of Genetics and Breeding in Forest Trees and Ornamental Plants, Ministry of Education, College of Biological Sciences and Technology, Beijing Forestry University, Beijing, 100083, China; d Department of Plant Physiology, Umeå Plant Science Centre (UPSC), Umeå University, Umeå, 90187, Sweden; e State Key Laboratory of Vegetable Biobreeding, Institute of Vegetables and Flowers, Chinese Academy of Agricultural Sciences, Beijing, 100081, China; f School of Life Sciences and Medicine, Shandong University of Technology, Zibo, 255000, China; g Key Laboratory of Crop Genetic Improvement & Ecology and Physiology, Institute of Crop Germplasm Resources, Shandong Academy of Agricultural Sciences, Ji'nan, 250100, China; h Guangdong Eco-Engineering Polytechnic, Guangzhou, 510520, China

**Keywords:** Allele identification, Allele-specific expression, Haplotype-resolved, Non-model diploid plant genome, Sequence similarity

## Abstract

Advanced sequencing now enables haplotype resolution of genomes from non-model diploid plant species, facilitating allele identification and the use of allele-specific expression (ASE) analysis to uncover the relationships between genes and phenotypes in heterozygous genomes. However, identification of true allelic pairs remains challenging due to the presence of paralogous genes from ancient genome duplications, and existing methods lack systematic, reproducible filtering criteria. In this study, we developed Alleleauto, a workflow integrating the parametric 3σ rule and the non-parametric Tukey's method as two complementary outlier detection methods to precisely identify alleles and perform ASE analysis from haplotype-resolved assemblies. Alleleauto first searches for homologous genes across homologous chromosomes, then applies statistical filtering criteria based on synonymous substitution rates (*Ks*) and synteny to systematically remove false alleles (paralogs). This dual-method framework offers flexible filtering strategies with adjustable parameters, enabling optimization for diverse genomes. We validated the workflow on tea plant (*Camellia sinensis*), ginger (*Zingiber officinale*), and lychee (*Litchi chinensis*), three plant species with distinct genomic features, demonstrating that statistical filtering significantly improves accuracy over the use of sequence similarity alone. Using the alleles identified by Alleleauto, we performed ASE analysis and calculated sequence divergence parameters to investigate ASE and heterosis mechanisms. Our open-source, and easy-to-use pipeline provides significant value for reproducible, scalable investigation of ASE and heterosis with haplotype-resolved genome assemblies.

*Dear Editor*,

Alleles are variant forms of genes located at the same position on homologous chromosomes. Each pair of alleles in a diploid genome represents the genotype of a specific gene. Identifying alleles is critical for understanding the potential relationship between genes and phenotypes [1] and further advancing molecular breeding for trait improvement. Allele-specific expression (ASE) refers to the preferential expression of one parental allele over the other in heterozygous plants [2,3], and ASE analysis is a powerful tool for studying the molecular genetics of alleles and for harnessing the power of heterosis. However, the quality of ASE analyses is highly dependent on the accuracy of allele identification.

Advances in long-read sequencing technologies have revolutionized genome assembly pipelines. This progress has yielded an increasing number of high-quality, haplotype-resolved telomere-to-telomere (T2T) reference genomes for non-model species, such as tea plant (*Camellia sinensis*) [4] and lychee (*Litchi chinensis*) [5]. Several strategies exist for the identification of different alleles from haplotype-resolved assemblies, such as aligning each haplotype assembly to a reference genome or identifying reciprocal best hits (RBH) gene pairs between haplotypes [6]. Current methods predominantly rely on sequence similarity and often neglect synteny information, which is essential for confirming allelic relationships. Importantly, a key limitation of current approaches is their inability to accurately distinguish different alleles of the same gene from different paralogs, particularly in complex plant genomes. True allelic pairs, which represent the same gene locus on homologous chromosomes, need to be differentiated from paralogous pairs that originated from whole-genome, segmental, or tandem duplications. These paralogs frequently exhibit high sequence similarity and can even retain syntenic relationships with the ancestral gene from which they originated, leading to their misidentification as alleles of a single gene instead of being distinct genes, especially in genomes with a history of polyploidization.

To address the challenge posed by complex plant genomes, we have developed Alleleauto, a work flow that integrates two complementary outlier detection methods: the parametric 3σ rule [7] for normally distributed data and the non-parametric Tukey's method [8] for non-normal distributions. By simultaneously applying statistical filters based on key metrics such as synonymous substitution rates (*Ks*) and chromosomal synteny, Alleleauto systematically discriminates true allelic pairs for paralogs. It further includes visualization modules for assessing allelic colinearity and ASE patterns, facilitating validation and functional analyses. We demonstrate the utility and superior performance of Alleleauto through its application to three case studies with distinct genomic backgrounds. Compared to existing ad hoc scripts and manual approaches, Alleleauto offers enhanced accuracy, reproducibility, and a user-friendly design.

Our pipeline involves two main steps (Fig. 1A). The first step is the identification of alleles within the haplotype-resolved genome assembly. Homologous gene pairs are first identified along homologous chromosomes based on chromosome correspondence information, protein sequences, coding sequences (CDS), and gene annotations from both haplotypes of a diploid genome assembly. Reciprocal best hits (RBH) are then selected from these homologous gene pairs using the sequence similarity survey method implemented in Genetribe v1.2.1 [9]. Subsequently, the syntenic block and sequence divergence (*Ka*, number of substitutions per non-synonymous site; *Ks*, number of substitutions per synonymous site; and *Ka*/*Ks*) of these gene pairs are calculated as implemented in WGDI v0.4.7 [10]. Next, a filtering step that examines colinearity removes homologous gene pairs that do not map to the same genetic locus between the two homologous chromosomes. Additionally, gene pairs with outlier *Ks* values are excluded using either the 3σ rule [7] or Tukey's method [8] (as detailed in the “Materials and Methods” section). The gene pairs that remain after the homologous survey and filtering step are considered to constitute true allele pairs (Fig. 1B). In this pipeline, allele identification and a dot plot testing the colinearity of the identified allele pairs can be achieved by a single-line command (see README.txt for details of each parameter). A genome colinearity dot plot for the identified allele pairs provides a convenient quality control method and enables straightforward manual adjustment if needed.

**Fig. 1:**
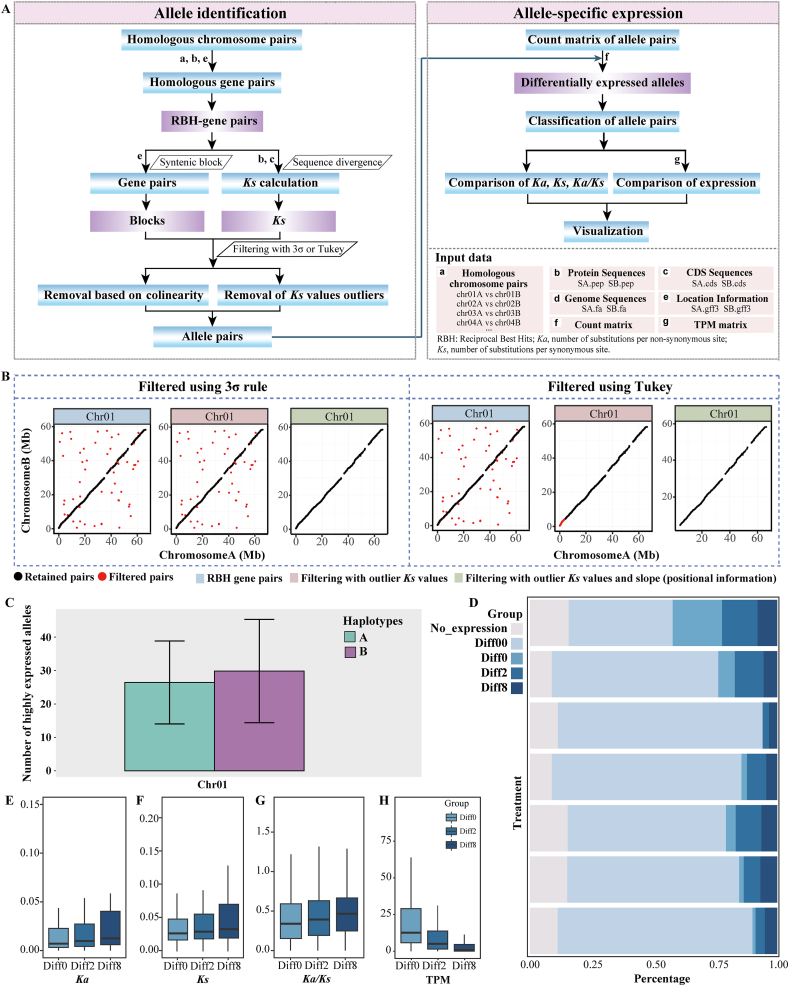
Workflow and example output of the Alleleauto pipeline. **A.** Workflow of the Alleleauto pipeline. **B.** Identification and visualization of alleles (with filtering following the 3σ rule and Tukey's method). **C.** Number of highly expressed genes from different homologous chromosomes in different haplotypes. Data are shown as means ± standard deviation (s.d.). **D.** Grouping of allele-specific expression (ASE) profiles among samples from different tissues and treatments. No_expression: Neither allele is expressed; Diff00: the pair of alleles are not significantly differentially expressed with *P*-adjust >0.05; Diff0: significant difference between a pair of alleles with *P*-adjust ≤0.05 and |fold-change (FC)| ≤ 2; Diff2: significant difference between a pair of alleles with *P*-adjust ≤0.05 and 2 < |FC| < 8; Diff8: significant difference between a pair of alleles with *P*-adjust ≤0.05 and |FC| ≥ 8. **E–G.** Boxplot visualization of *Ka*, *Ks*, and the *Ka*/*Ks* ratio for each allele pair from the three differentially expressed categories (Diff0, Diff2, and Diff8). The centerline represents the 50th percentile. The whiskers indicate the minimum and maximum values. **H.** Absolute difference in gene expression (in TPM) for the three differentially expressed categories of allele-specific gene expression.

The second step compares the expression of paired alleles to characterize ASE patterns (Fig. 1A). Differential expression is computed based on gene expression count matrices using the DESeq2 package [11], requiring at least three biological replicates per treatment by default. For each allele pair, fold-change (FC) values and statistical significance are calculated, after which allele pairs are classified into five ASE categories based on expression patterns: (1) No_expression, when neither allele is expressed; (2) Diff00, when they are not significantly differentially expressed (*P*-adjust >0.05); (3) Diff0, when they are significantly differentially expressed with *P*-adjust ≤0.05 but |FC| ≤ 2; (4) Diff2, when they are significantly differentially expressed with *P*-adjust ≤0.05 and 2 < |FC| < 8; and (5) Diff8, when they are significantly differentially expressed with *P*-adjust ≤0.05 and |FC| ≥ 8 (Fig. 1D). This pipeline can also be applied to calculate gene expression levels, *Ka*, *Ks*, and the *Ka*/*Ks* ratio for allele pairs across different comparison groups (Fig. 1E–H). Gene expression patterns across these groups can be examined and visualized using the command: “bash allele_specific_expression.sh”.

To systematically evaluate the performance and applicability of the Alleleauto pipeline, we performed testing using real genomic datasets from three evolutionarily divergent plant species, namely tea plant (*Camellia sinensis*), ginger (*Zingiber officinale*), and lychee (*Litchi chinensis*). These three sample datasets are derived from diverged plant lineages with distinct genomic characteristics, including genome size, ancient whole-genome duplication (WGD), and segmental duplication (Table S1).

(1) Tea dataset. In the tea plant dataset (3.06 Gb for haplotype A, 2.92 Gb for haplotype B, heterozygosity of 2.3%), we identified 13,840 RBH gene pairs, of which 12,138 were located within colinear blocks (Fig. S1 and Table S2). The 3σ rule excluded 25 gene pairs with anomalous *Ks* values and off-diagonal positions, yielding 12,113 high-confidence allele pairs (Table S2). For comparison, Tukey's method (default parameter i = 1.5) retained only 10,333 allele pairs, demonstrating greater stringency (Fig. S1 and Table S2). Genome colinearity dot plots confirmed that both methods effectively preserved syntenic structure while removing outliers, thereby distinguishing paralogs from true alleles. The 3σ rule successfully eliminated most false-positive pairs, producing sharp diagonal patterns with minimal off-diagonal noise. Although Tukey's method provided additional stringency, it may be overly conservative for the tea plant genome, potentially excluding numerous genuine allele pairs. For a diploid species like tea plant with low heterozygosity (2.3%) and near-normal *Ks* distributions, the 3σ rule effectively balanced true allele retention with removal of false-positives. Tukey's method may be overly stringent, excluding many genuine allele pairs. We therefore recommend the 3σ rule for genomes with similar characteristics to the tea plant genome.
(2) Ginger dataset. The ginger genome (1.6 Gb for haplotype A, 1.4 Gb for haplotype B, heterozygosity of 3.6%) presents greater complexity due to its polyploid history, comprising three events: the *Musa*-shared whole-genome duplication (WGD, ∼63 Mya), and a ginger-specific WGD (∼27 Mya). From 26,470 RBH gene pairs, 24,526 were co-localized within colinear blocks (Fig. S2 and Table S2). The 3σ rule removed five outlier pairs, while Tukey's method (i = 1.5) eliminated 4934 pairs (Fig. S2 and Table S2). Colinearity plots confirmed that both methods effectively revealed primary syntenic structures between two haplotypes. The polyploid history of ginger produces a wide *Ks* distribution, which may help explain why the 3σ filter removed only five outliers, in contrast to nearly 5000 gene pairs with Tukey's method. For comprehensive allele discovery, the 3σ rule is preferable; for a high-confidence allele set for evolutionary analysis, Tukey's method is more suitable.
(3) Lychee dataset. The lychee genome (450 Mb for haplotype A, 455 Mb for haplotype B, heterozygosity of 2.3 %) has not undergone a WGD since the ancient gamma triplication (∼117 Mya). Among 21,780 RBH gene pairs, 19,970 were located within colinear blocks (Table S2). However, the 3σ rule failed to eliminate all false positives. Indeed, colinearity mapping revealed residual non-diagonal blocks (red circle) representing undetected paralogous pairs (Fig. S3), highlighting a key limitation of parametric filtering when data distributions deviate from normality or when genome-specific factors (e.g., large-scale segmental duplications independent of WGD) introduce complexity. Systematic testing with Tukey's method across varying Interquartile Range (IQR) parameters (i = 1.5 to 8) demonstrated progressively improved performance (Fig. S4–S11). At i = 8, non-diagonal false-positive gene pairs were eliminated while substantially recovering putative allelic pairs along chromosome 14 (Fig. S11). This observation demonstrates the flexibility and controllability of Tukey's method through parameter optimization. For genomes with non-normal *Ks* distributions, we recommend a tunable non-parametric method like Tukey's.
(4) Comparison with AlleleFinder. To validate the performance of Alleleauto against an existing tool, we chose AlleleFinder v1.2.1 (https://github.com/sc-zhang/AlleleFinder) and employed both tools on the same three species (Tables S3 and S4). AlleleFinder uses a multi-step pipeline (BLAST + MCScanX, GMAP, iterative refinement, to name a few) and was run with the parameters --is_mono (specifying a monoploid genome), -n 2 (setting the ploidy level to 2), and -t 8 (using eight threads). In terms of computational resources (Table S3), Alleleauto used ∼1 GB RAM across all datasets, while AlleleFinder required 3–24 GB (up to 23.65 GB for tea plant). AlleleFinder required fewer total CPU hours (0.46–3.50 h), reflecting due to its lightweight design and low core usage (2–3 cores). However, on large genomes like that of tea plant, Alleleauto achieved better wall-clock performance by exploiting high parallelism (12–15 cores). In terms of result accuracy, both methods showed high consistency across all species, sharing 10,538–28,257 allele pairs (Table S4). Overlap with Alleleauto output was 87.2–97.2%, whereas overlap with AlleleFinder output was 58.2–96.6%, indicating that AlleleFinder generates a larger, more inclusive set. Overall, Alleleauto applied a stricter filtering strategy, producing a smaller but higher-confidence set, while AlleleFinder provided broader coverage at the cost of higher memory usage.

We performed ASE analysis to investigate the possible divergence in expression of allelic gene pairs in tea plant and ginger. Analysis of allele expression across homologous chromosome pairs revealed no significant haplotype-level bias in transcript levels between allele pairs in either tea plant or ginger (Figs. S12A and S13A). However, we did observe widespread ASE in both species. The Diff00 category (no significant different between pairs) represented the highest proportion of expressed allelic gene pairs, while Diff8 (significant difference with absolute |fold-change| ≥ 8) was consistently the least abundant across both species (Figs. S12B and S13B). To examine the relationship between sequence evolution and expression divergence, we compared nucleotide substitution rates among ASE categories. Allele pairs in the Diff8 category exhibited significantly higher *Ka*, *Ks*, and *Ka*/*Ks* values than those in the other categories for both tea plant and ginger (Fig. S12C–E and S13C–E), suggesting that greater sequence divergence may contribute to pronounced expression differences. Furthermore, allele pairs in the Diff0 category (significant difference with |FC| ≤ 2) showed significantly higher absolute expression levels than those in the Diff2 and Diff8 categories in both datasets (Figs. S12F and S13F).

In summary, we developed Alleleauto, an automated workflow that combines the 3σ rule, Tukey's outlier detection, and colinearity analysis to accurately identify allelic pairs. Unlike traditional tools that rely on fixed similarity thresholds, Alleleauto establishes a statistical classification framework that integrates evolutionary divergence with positional colinearity, thereby distinguishing true alleles from paralogs. Alleleauto offers several advantages compared to the existing software AlleleFinder, including low memory consumption, stable performance on large-scale genomes, and excellent scalability, enabling it to run efficiently on standard computing resources. Validation across multiple species, including an interspecific hybrid diploid [12], a polyploid [13], and a genome with extremely low heterozygosity [14], demonstrates that its flexible filtering strategy can adapt to the diverse evolutionary patterns of plant genomes. Furthermore, ASE analysis conducted using Alleleauto revealed positive correlations between sequence divergence and expression differences, providing insight into allele evolution and regulation of gene expression. As a valuable open-source tool, Alleleauto enables reproducible, accurate, and high-throughput allele identification, which will significantly advance future research into plant allelic variation, expression differentiation, and molecular breeding.

## 1 Materials and methods

### 1.1 Data collection

Genome assembly and annotation information were acquired for tea plant (*C. sinensis*) from the China National Center for Bioinformation under accession number GWHASIX00000000 [4], for ginger (*Z. officinale*) under the NCBI BioProject ID PRJNA647255 [15], and for lychee (*L. chinensis*) from Mendeley data (https://data.mendeley.com/datasets/kggzfwpdr9/1) [5]. Furthermore, 108 Gb of RNA-seq data were downloaded for tea plant from the NCBI Short Read Archive (SRA) database (project ID PRJNA665594), representing 18 samples across six different tissues and three replicates per tissue [5] (Table S5). RNA-seq data for ginger were downloaded from the NCBI SRA database under project ID PRJNA647255, comprising 21 samples and a total of seven tissues, each with three replicates [15]. ASE analysis was not performed on the lychee data due to the absence of replicates in the design of available RNA-seq datasets.

### 1.2 RNA-seq data processing

Fastp v0.20.1 [16] was employed for preprocessing the collected Illumina RNA-seq reads, by removing adapters and the low-quality bases (30 < score). The clean reads were then mapped against the respective genome assembly using HISAT2 v2.1.0 (https://github.com/infphilo/hisat2) with default settings [17]. Subsequently, the mapped reads were retained to calculate the counts and transcripts per kilobase million (TPM) matrices with featureCounts [18].

### 1.3 Allele identification using the 3σ rule

The initial step of the Alleleauto pipeline involves the identification of alleles within the haplotype-resolved genome assembly (Fig. 1). Homologous gene pairs between two haplotypes were identified using protein sequences, coding sequences, and gene annotations (GFF format). Gene pairs meeting the RBH criterion were selected using Genetribe v1.2.1 [9]. To remove false-positive pairs, the 3σ rule was applied, which assumes that *K*s values follow a normal distribution and defines outliers as observations falling beyond three standard deviations (σ) from the mean (μ): *Ks* < μ − 3σ or *Ks* > μ + 3σ [7]. This parametric approach is suitable for genomes with relatively simple duplication histories where *Ks* values approximate a normal distribution.

### 1.4 Allele identification using Tukey's method

RBH gene pairs were obtained using the previously described method. To identify and remove outliers in the dataset, Tukey's method was employed [8] a distribution-free approach based on quartiles.

In this method, *Q1* and *Q3* represent the 25th and 75th percentiles, respectively. The interquartile range (IQR) is calculated as:

IQR = *Q3* − *Q1*

Outliers are defined using adjustable fences:

Inner fences:

[*Q1* − i × IQR, *Q3* + i × IQR]

Outer fences:

[*Q1* − 2i × IQR, *Q3* + 2i × IQR]

Values beyond the chosen fence were classified as outliers [19]. A smaller i value narrows the acceptance window, removing more pairs (stricter), whereas a larger i value widens the window, removing fewer pairs (more permissive). A customized outlier detection function was developed in the R programming environment to implement this method, which was then applied to filter detected outliers from the RBH gene pairs. This process ensured robustness and reliability in the identification of gene pairs and determination of differential expression.

### 1.5 Integration with synteny information

Beyond *Ks*-based filtering, chromosomal position information was incorporated to ensure that the identified alleles maintain syntenic relationships. True allelic pairs should occupy corresponding positions along homologous chromosomes, forming clear colinear blocks. After initial *Ks*-based removal of outliers, the slope of gene positions between homologous chromosomes was calculated for each syntenic block and blocks with aberrant slope values were removed using the same statistical framework. This dual-layer filtering based on *Ks* values first, then on positional relationships, provides stringent quality control while maintaining biological relevance.

### 1.6 Allele-specific gene expression (ASE) analysis

ASE analysis is the second part of the Alleleauto pipeline (Fig. 1). To identify differentially expressed allele pairs, the “DESeq2” package was applied [11]; by default, three replicates per treatment were included. Subsequently, allele pairs were classified into distinct groups based on their fold-change (FC) values and significance of difference, resulting in the formation of five ASE groups: No_expression, Diff00, Diff0, Diff2, and Diff8.

## CRediT authorship contribution statement

**Tian-Le Shi:** Writing – original draft, Data curation, Conceptualization. **Shuai Nie:** Data curation, Conceptualization. **Yu-Tao Bao:** Methodology, Data curation. **Zhi-Chao Li:** Methodology, Formal analysis. **Zhao-Yang Chen:** Data curation. **Shi-Wei Zhao:** Data curation. **Xue-Mei Yan:** Software. **Hai-Yao Ma:** Data curation. **Xue-Chan Tian:** Software. **Kai-Hua Jia:** Methodology, Data curation. **Jing-Fang Guo:** Writing – original draft. **Jun-Ke Zhang:** Writing – review & editing. **Jian-Feng Mao:** Writing – review & editing, Conceptualization.

## Declaration of competing interest

The authors declare that they have no known competing financial interests or personal relationships that could have appeared to influence the work reported in this paper.

## Data Availability

The pipeline, exemplar data, and user guide are publicly available at https://github.com/shitianle77/Alleleauto.
