# Supplementary material for: Alleleauto: a pipeline for allele identification and analysis of allele-specific gene expression with haplotype-resolved diploid genome assemblies
Source: aBIOTECH. 2026 May 19;7(3):100056. doi: 10.1016/j.abiote.2026.100056 (PMC13240741; doi:10.1016/j.abiote.2026.100056)
Supplement: Multimedia component 1 [file mmc1.docx]

**Table S1. Summary of genomic characteristics for species analyzed in this work.**

| **Species** | ***Camellia sinensis*** | ***Zingiber officinale*** | ***Litchi chinensis*** |
| --- | --- | --- | --- |
| **Genome size** | Hap A: 3.06 Gb  Hap B: 2.92 Gb | Hap A: 1.6 Gb  Hap B: 1.4 Gb | Hap A: 450 Mb  Hap B: 455 Mb |
| **Total** **gene number** | Hap A: 32,596 Hap B: 24,723 | Hap A: 35,883 Hap B: 35,395 | Hap A: 27,687 Hap B: 27,711 |
| **Repeat content (%)** | Hap A: 74.3  Hap B: 74.2 | ~56.5 | Hap A: 56.6  Hap B: 56.48 |
| **Heterozygosity (%)** | 2.3 | ~3.6 | 2.3 |
| **WGD** | Ancient WGD event shared with Ericales relatives | Three polyploidization events: *Musa*-shared WGD, ginger-specific WGD | No WGD after the ancient γ triplication event |

**Table S2. Summary statistics of alleles filtered using the 3σ rule or Tukey’s method.**

| **Outlier filtering method** | **Number of gene pairs** | **Tea**  **(*Camellia sinensis*)** | **Ginger**  **(*Zingiber officinale*)** | **Lychee**  **(*Litchi chinensis*)** |
| --- | --- | --- | --- | --- |
| 3σ rule | Number of RBH gene pairs | 13,840 | 26,470 | 21,780 |
|  | Number of potential allele pairs on syntenic blocks | 12,138 | 24,526 | 19,970 |
|  | Number of remaining allele pairs after filtering outliers with *Ks* values | 12,114 | 24,521 | 19,969 |
|  | Number of remaining allele pairs after filtering with outlier *Ks* values and slope (positional information) | 12,114 | 24,521 | 19,788 |
| Tukey’s method | Number of RBH gene pairs | 13,840 | 26,470 | 21,780 |
|  | Number of potential allele pairs on syntenic blocks | 12,138 | 24,526 | 19,970 |
|  | Number of remaining allele pairs after filtering outliers with *Ks* values | 10,682 | 21,482 | 19,849 |
|  | Number of remaining allele pairs after filtering with outlier *Ks* values and slope (positional information) | 10,333 | 19,592 | 19,204 |

Note: RBH, reciprocal best hit.

**Table S3. Comparison of runtime performance between Alleleauto and AlleleFinder across different species.**

| **Species** | **Tool** | **Wall time (h)** | **CPU hours** | **Peak memory (GB)** |
| --- | --- | --- | --- | --- |
| *Camellia sinensis* | Alleleauto | 0.62 | 9.34 | 0.97 |
|  | AlleleFinder | 1.23 | 3.5 | 23.65 |
| *Zingiber officinale* | Alleleauto | 1.19 | 14.95 | 1.08 |
|  | AlleleFinder | 0.76 | 2.64 | 8.62 |
| *Litchi chinensis* | Alleleauto | 0.94 | 13.36 | 0.94 |
|  | AlleleFinder | 0.18 | 0.46 | 3.89 |
| *Populus* 84K | Alleleauto | 1.26 | 15.86 | 1.08 |
|  | AlleleFinder | 0.19 | 0.56 | 3.13 |
| *Coriaria nepalensis* | Alleleauto | 1.05 | 11.46 | 1.24 |
|  | AlleleFinder | 0.12 | 0.24 | 3.57 |
| *Triticum aestivum* | Alleleauto | 1.22 | 18.22 | 3.22 |
|  | AlleleFinder | 2.16 | 6.41 | 22.5 |

Note: CPU, central processing unit.

**Table S4. Comparison of allele identification results from Alleleauto and AlleleFinder.**

| **Species** | **Method** | **Alleleauto** | **AlleleFinder** | **Shared** | **Recall vs Alleleauto** | **Recall vs AlleleFinder** |
| --- | --- | --- | --- | --- | --- | --- |
| *Camellia sinensis* | 3σ rule | 12,083 | 18,104 | 10,538 | 87.2% | 58.2% |
| *Zingiber officinale* | 3σ rule | 24,584 | 28,499 | 21,668 | 88.1% | 76.0% |
| *Litchi chinensis* | Tukey’s method (i=8) | 17,931 | 25,158 | 17,435 | 97.2% | 69.3% |
| Hybrid poplar 84K | 3σ rule | 24,531 | 28,693 | 21,968 | 89.6% | 76.6% |
| *Coriaria nepalensis* | 3σ rule | 29,155 | 29,240 | 28,257 | 96.9% | 96.6% |
| *Triticum aestivum* | Tukey’s method (i=1) | 20,318 | 34,126 | 16,577 | 81.6% | 48.6% |

Notes: Recall versus Alleleauto was computed as |shared| / |Alleleauto total|; recall versus AlleleFinder was computed as |shared| / |AlleleFinder total|.

**Table S5. Summary statistics of RNA-seq samples from the tea plant dataset.**

| **Raw reads** | **Project ID** | **Sample name** | **Sequencing platform** | **Total bases (Gb)** | **Data size (Gb)** | **Mapping rate (%)** |
| --- | --- | --- | --- | --- | --- | --- |
| SRR12744831 | PRJNA665594 | RNAseq-stem-1 | Illumina HiSeq 2500 | 5.7 | 1.7 | 93.2 |
| SRR12744830 | PRJNA665594 | RNAseq-stem-2 | Illumina HiSeq 2500 | 5.6 | 1.7 | 92.3 |
| SRR12744829 | PRJNA665594 | RNAseq-stem-3 | Illumina HiSeq 2500 | 6.9 | 2.1 | 93.6 |
| SRR12744835 | PRJNA665594 | RNAseq-ol-1 | Illumina HiSeq 2500 | 6.2 | 1.9 | 93.6 |
| SRR12744834 | PRJNA665594 | RNAseq-ol-2 | Illumina HiSeq 2500 | 5.2 | 1.6 | 93.4 |
| SRR12744832 | PRJNA665594 | RNAseq-ol-3 | Illumina HiSeq 2500 | 6.2 | 1.9 | 93.9 |
| SRR12744838 | PRJNA665594 | RNAseq-yl-1 | Illumina HiSeq 2500 | 5 | 1.5 | 93.8 |
| SRR12744837 | PRJNA665594 | RNAseq-yl-2 | Illumina HiSeq 2500 | 5.1 | 1.5 | 93.5 |
| SRR12744836 | PRJNA665594 | RNAseq-yl-3 | Illumina HiSeq 2500 | 6 | 1.8 | 94.1 |
| SRR12744841 | PRJNA665594 | RNAseq-root-1 | Illumina HiSeq 2500 | 5.5 | 1.7 | 85.0 |
| SRR12744840 | PRJNA665594 | RNAseq-root-2 | Illumina HiSeq 2500 | 5 | 1.5 | 85.2 |
| SRR12744839 | PRJNA665594 | RNAseq-root-3 | Illumina HiSeq 2500 | 6.2 | 1.8 | 85.5 |
| SRR12744822 | PRJNA665594 | RNAseq-bud-1 | Illumina HiSeq 2500 | 5.8 | 1.7 | 93.3 |
| SRR12744843 | PRJNA665594 | RNAseq-bud-2 | Illumina HiSeq 2500 | 5.5 | 1.6 | 93.2 |
| SRR12744842 | PRJNA665594 | RNAseq-bud-3 | Illumina HiSeq 2500 | 6.9 | 2.1 | 93.2 |
| SRR13223884 | PRJNA665594 | RNAseq-fl-1 | Illumina HiSeq 2500 | 7.1 | 2.1 | 91.1 |
| SRR13223883 | PRJNA665594 | RNAseq-fl-2 | Illumina HiSeq 2500 | 7.1 | 2.1 | 91.2 |
| SRR13223882 | PRJNA665594 | RNAseq-fl-3 | Illumina HiSeq 2500 | 7.1 | 2 | 91.4 |
