# Supplementary material for: Alleleauto: a pipeline for allele identification and analysis of allele-specific gene expression with haplotype-resolved diploid genome assemblies
Source: aBIOTECH. 2026 May 19;7(3):100056. doi: 10.1016/j.abiote.2026.100056 (PMC13240741; doi:10.1016/j.abiote.2026.100056)
Supplement: Multimedia component 2 [file mmc2.docx]

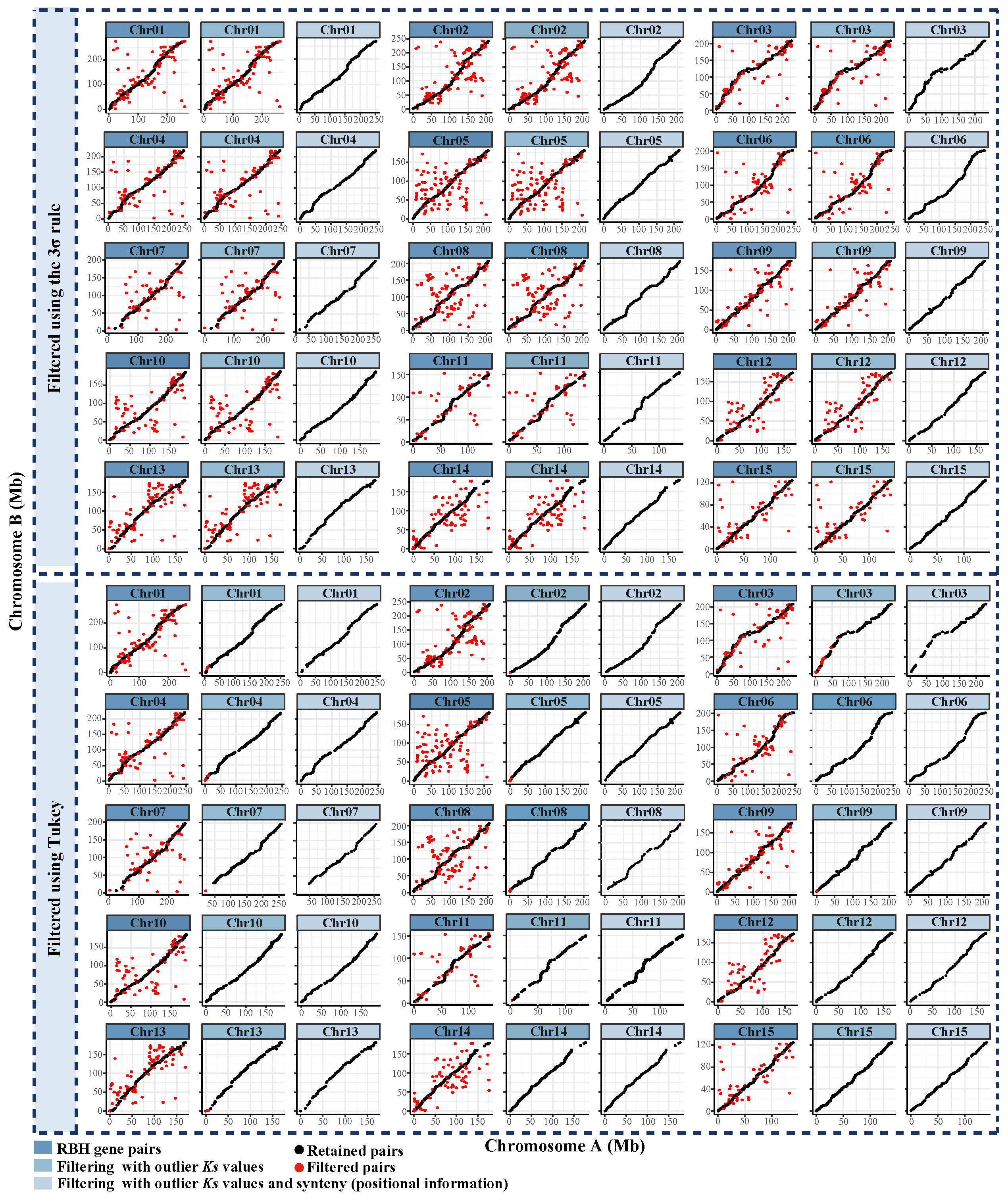


**Fig. S1.** Identification and visualization of alleles in the tea plant (*Camellia sinensis*) dataset. RBH, reciprocal best hits.


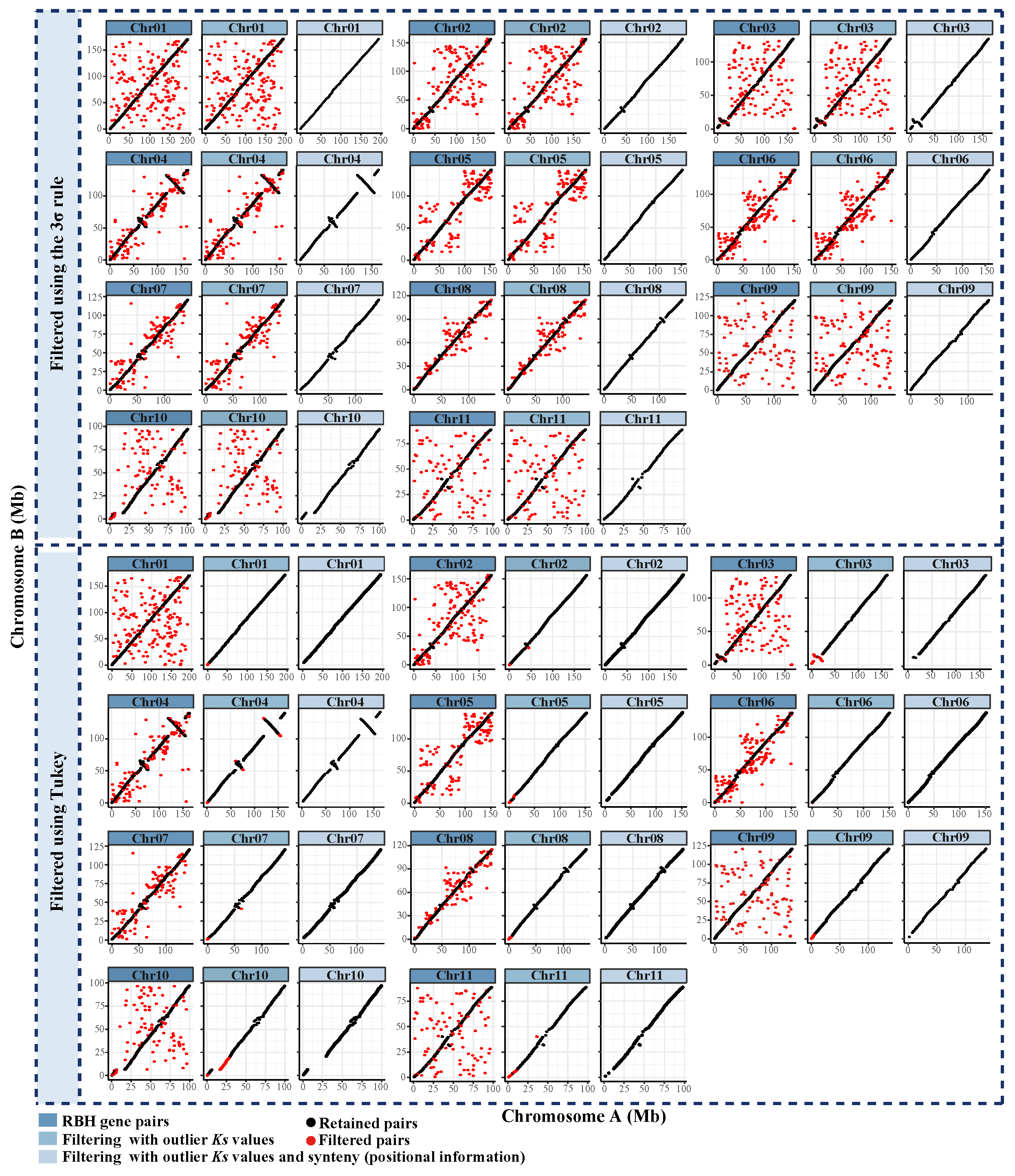


**Fig. S2.** Identification and visualization of alleles in the ginger (*Zingiber officinale*) dataset. RBH, reciprocal best hits.


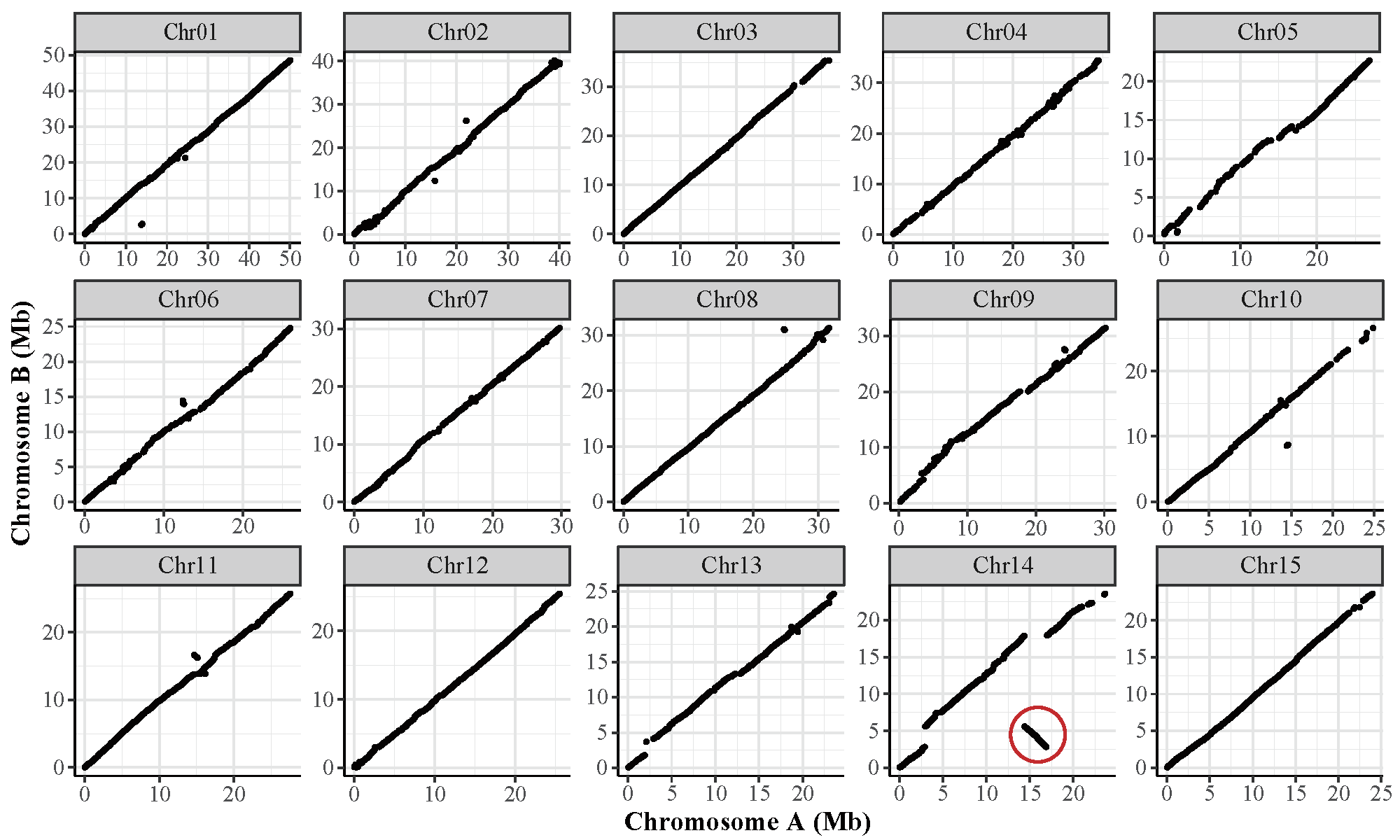


**Fig. S3.** Synteny blocks of identified allele pairs in lychee (*Litchi. chinensis*) using the 3σ rule. The colinear block within the red circle represents the colinear block where non-allelic genes are located.


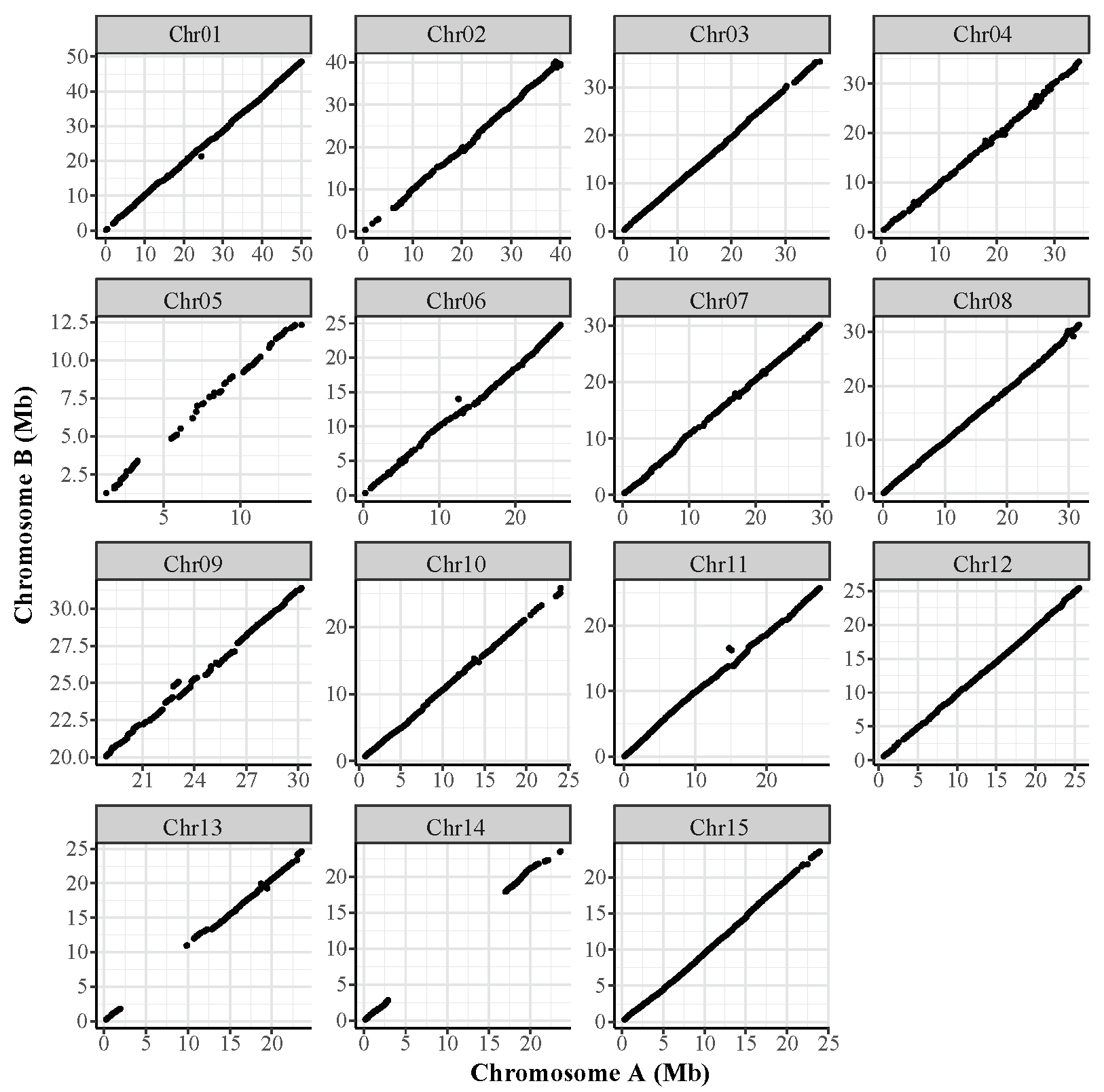


**Fig. S4.** Synteny blocks of identified allele pairs in lychee using Tukey’s method with 1.5 interquartile range (IQR).


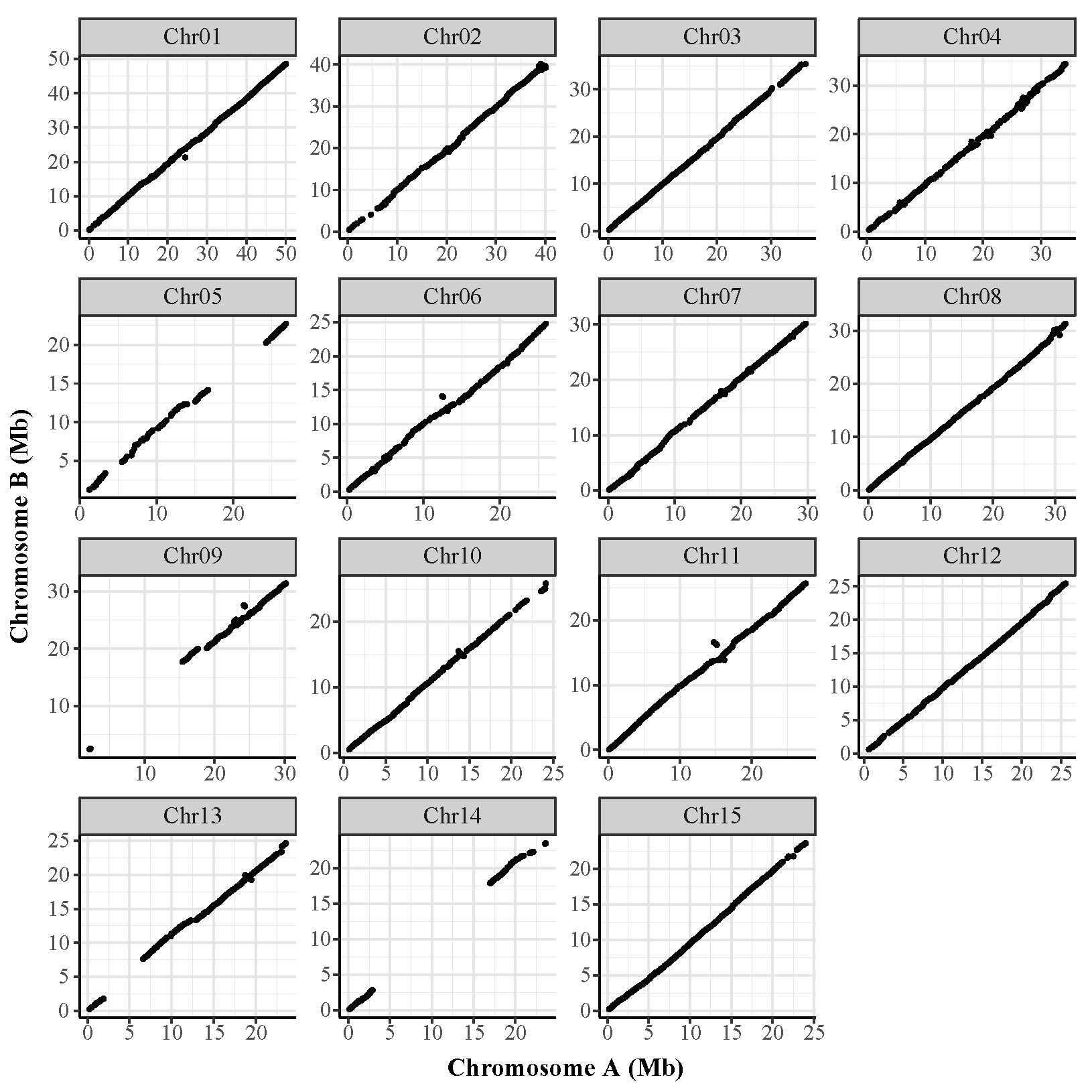


**Fig. S5.** Synteny blocks of identified allele pairs in lychee using Tukey’s method with 2 IQR.


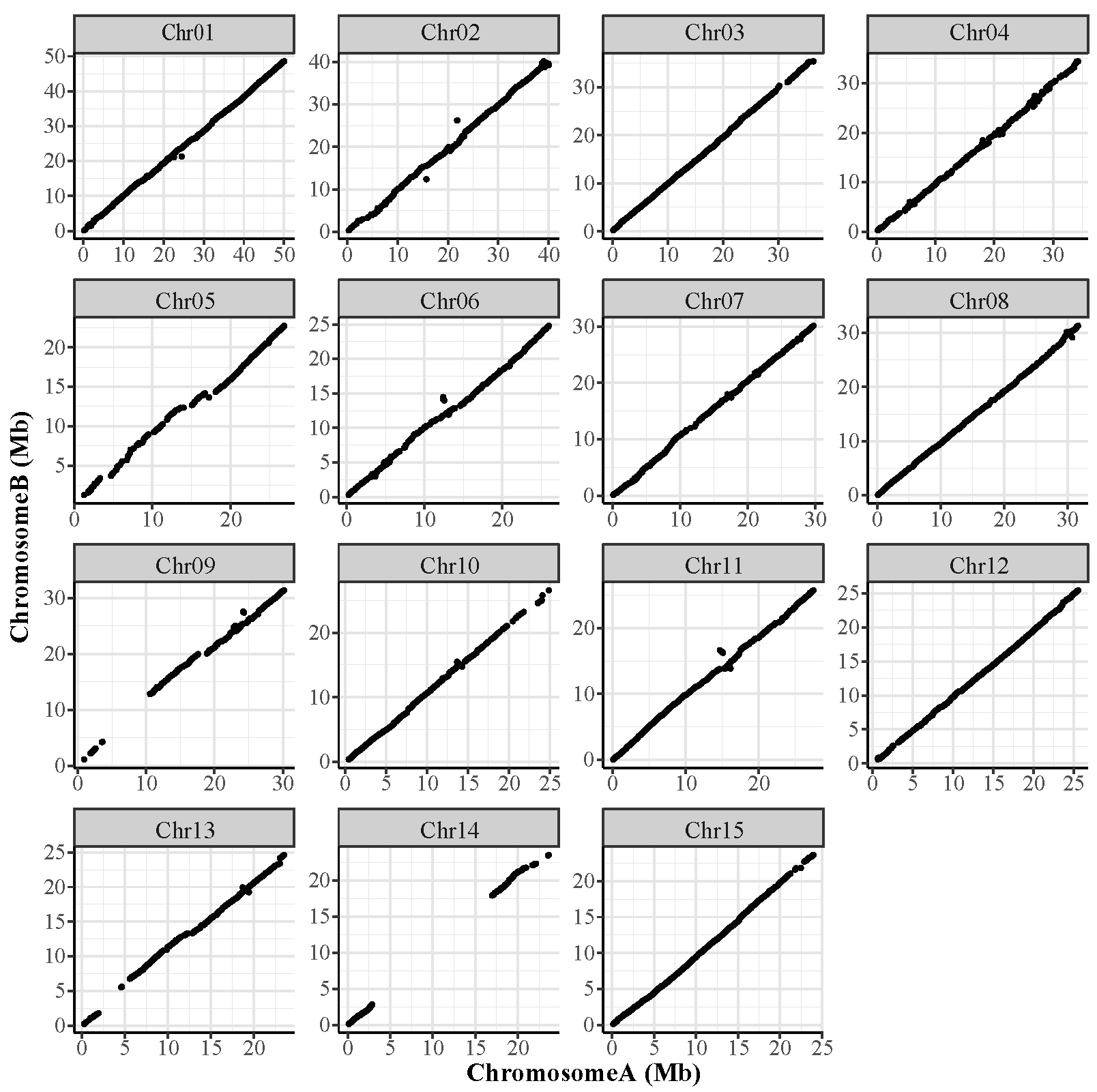


**Fig. S6.** Synteny blocks of identified allele pairs in lychee using Tukey’s method with 3 IQR.


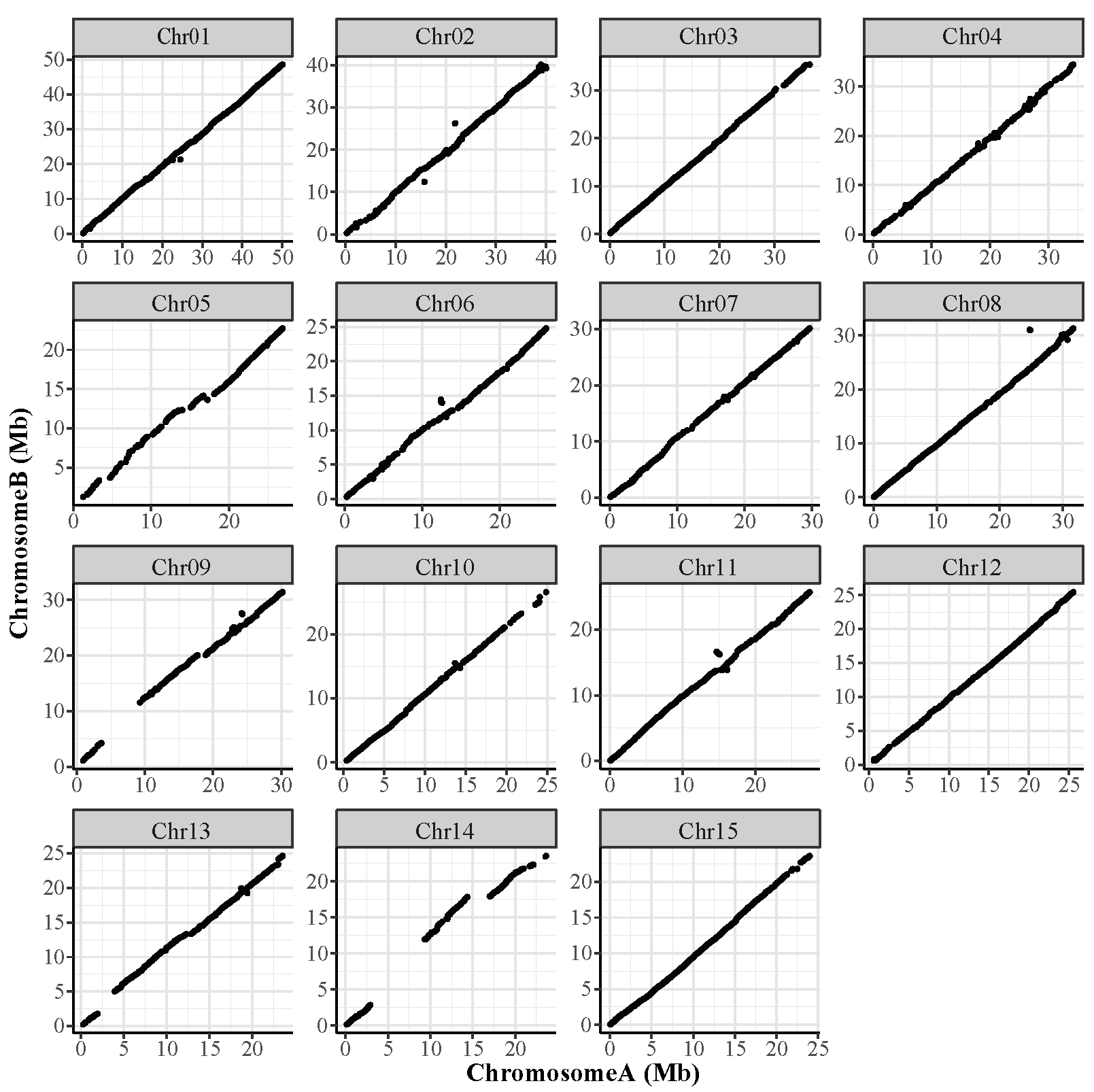


**Fig. S7.** Synteny blocks of identified allele pairs in lychee using Tukey’s method with 4 IQR.


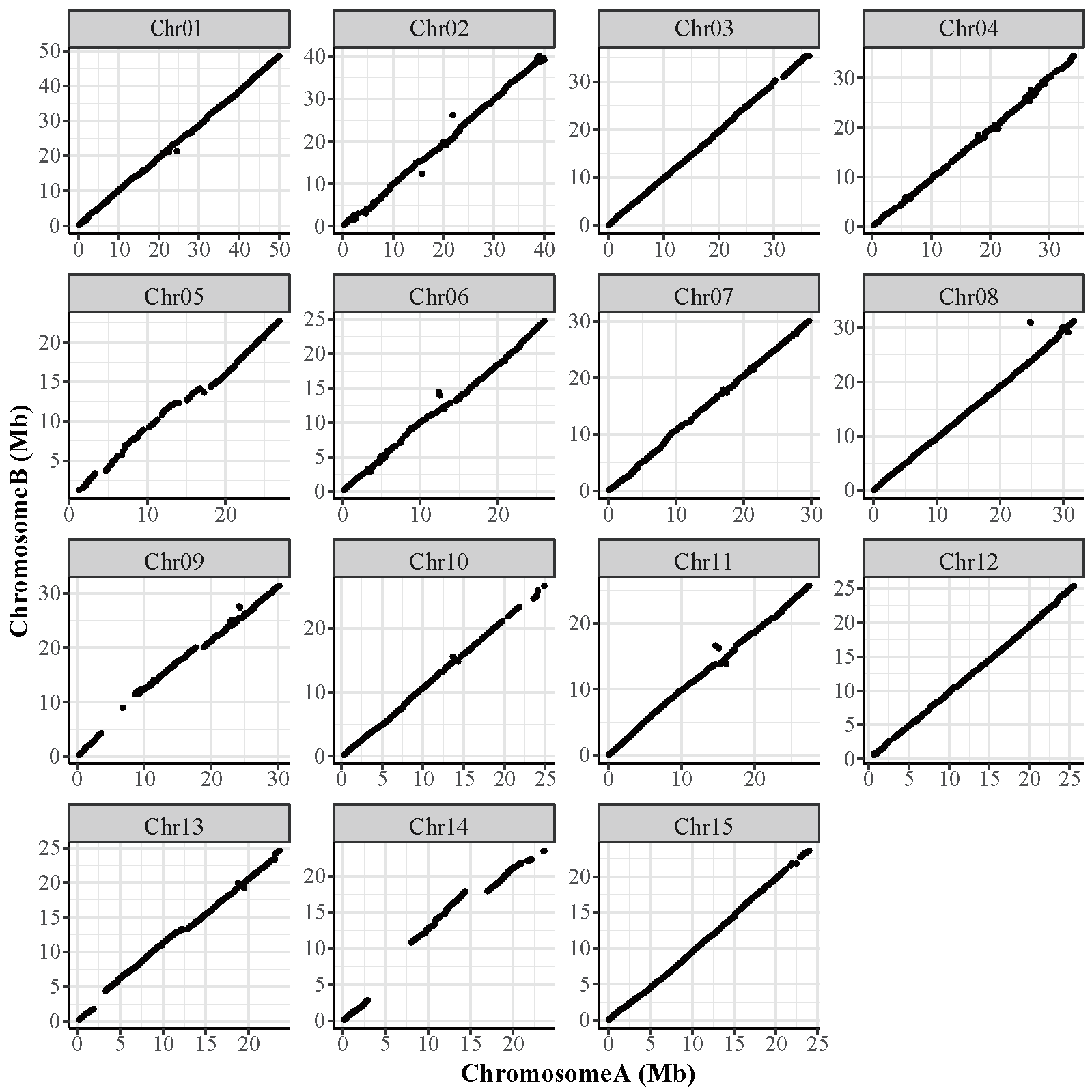


**Fig. S8.** Synteny blocks of identified allele pairs in lychee using Tukey’s method with 5 IQR.


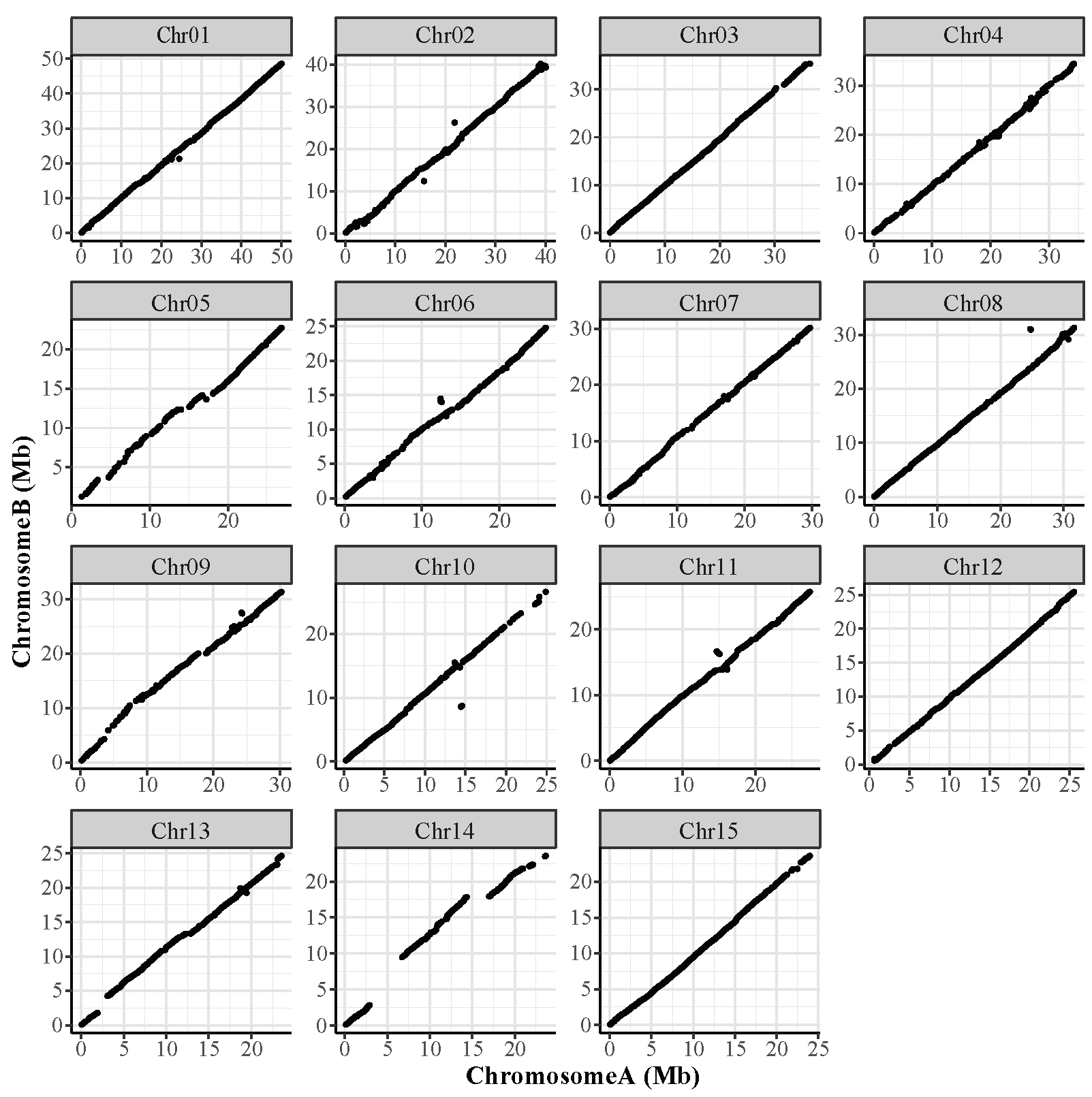


**Fig. S9.** Synteny blocks of identified allele pairs in lychee using Tukey’s method with 6 IQR.


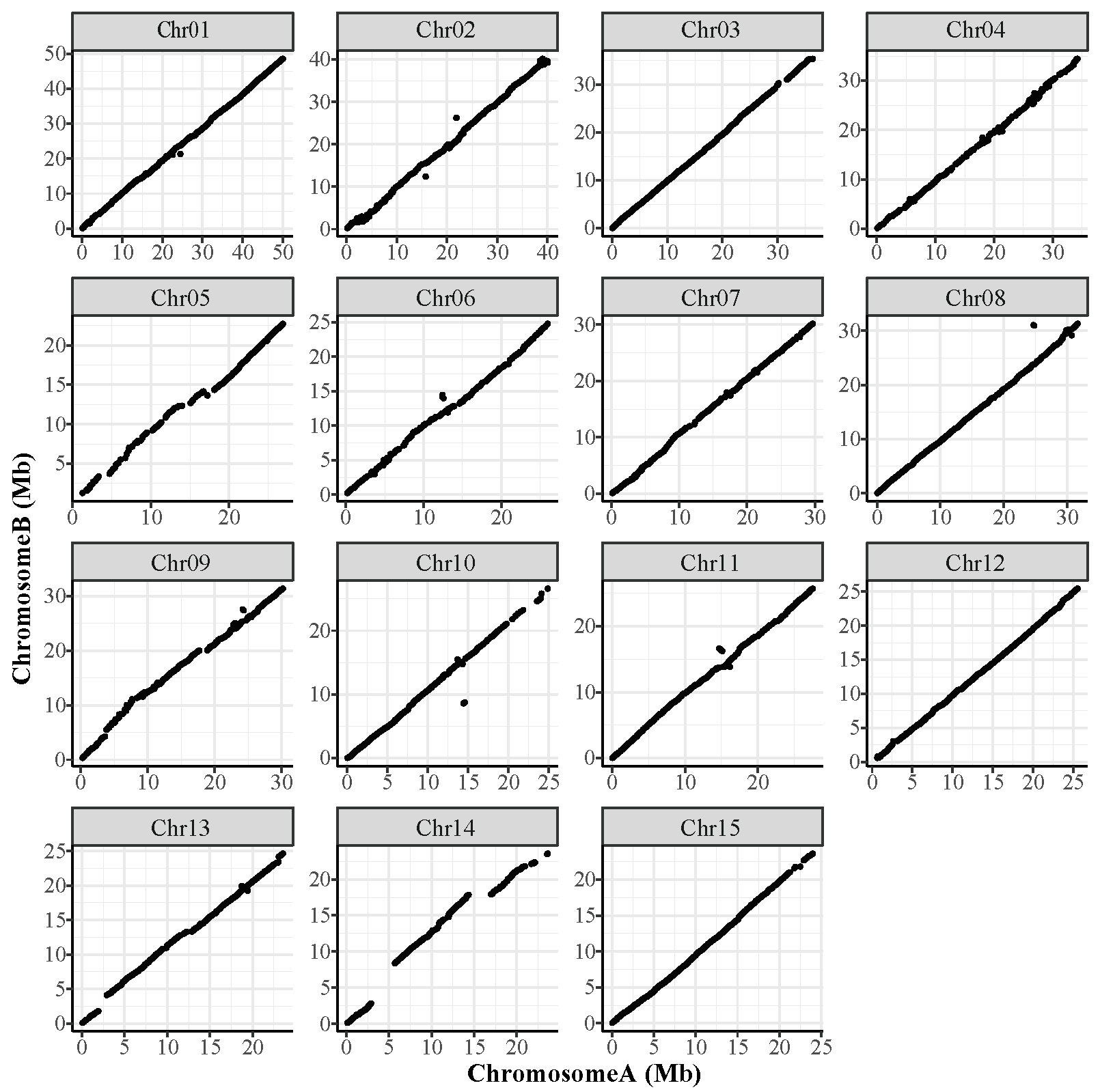


**Fig. S10.** Synteny blocks of identified allele pairs in lychee using Tukey’s method with 7 IQR.


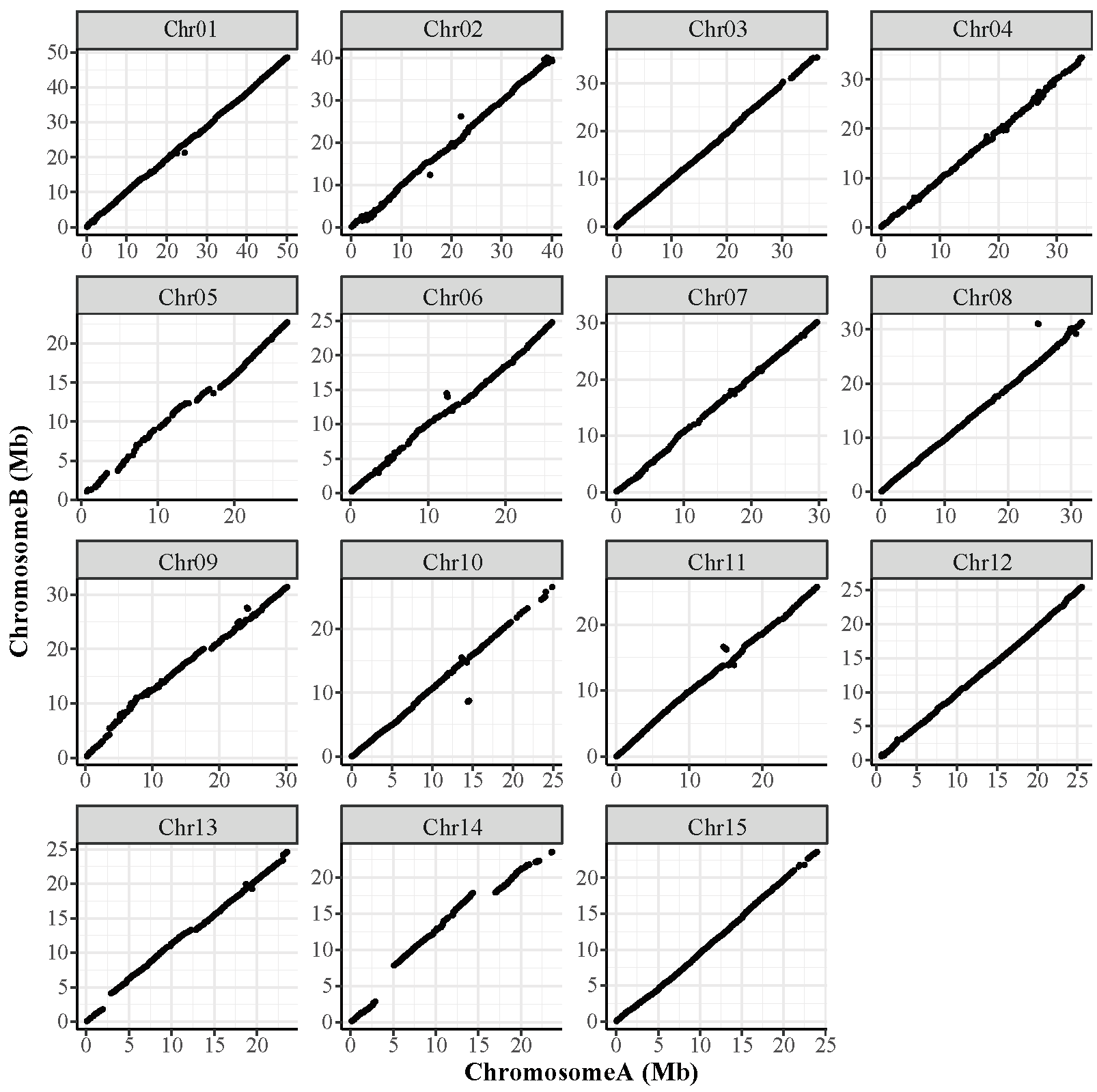


**Fig. S11.** Synteny blocks of identified allele pairs in lychee using Tukey’s method with 8 IQR.


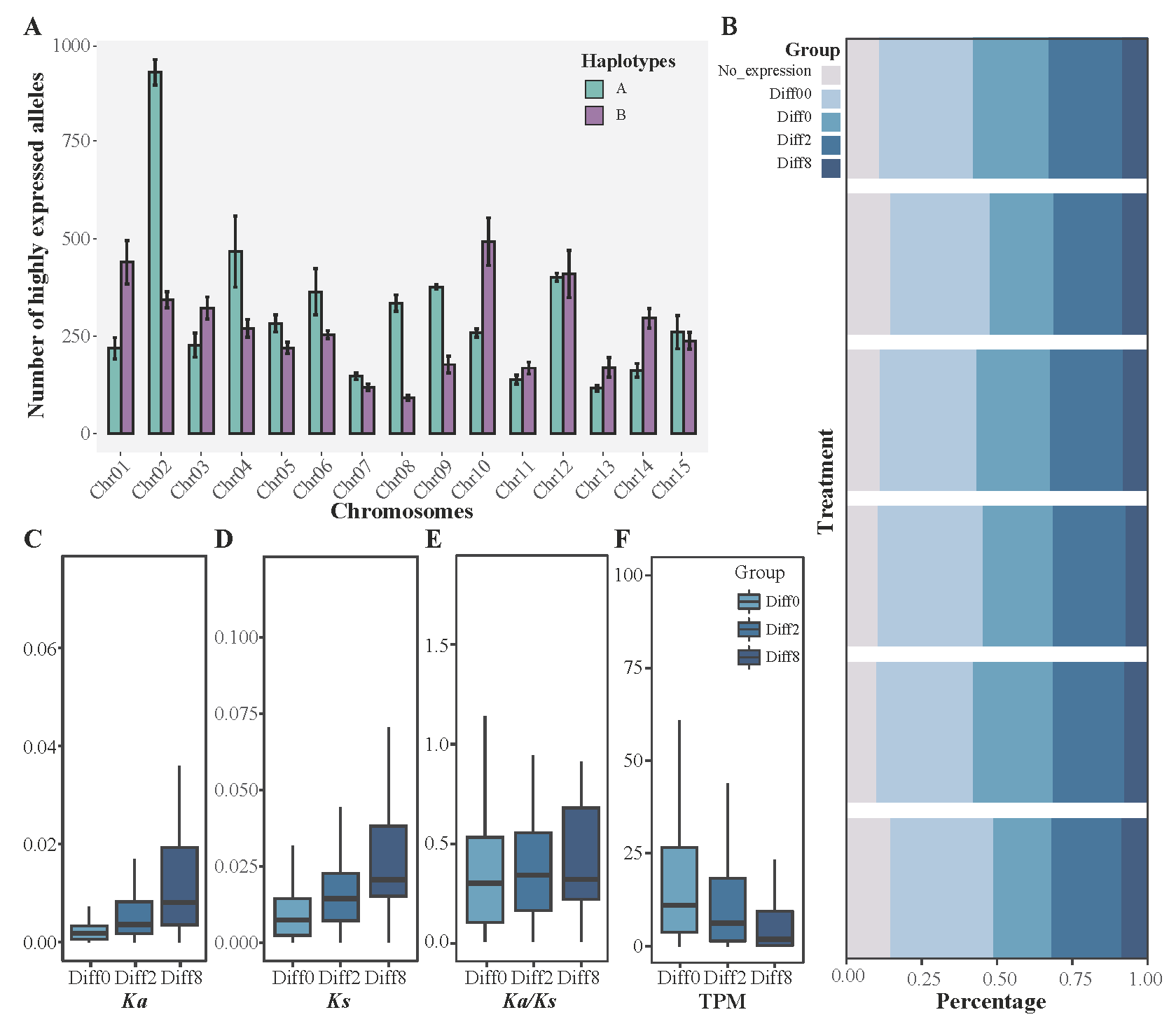


**Fig. S12.** Allele-specific expression (ASE) analysis in the tea plant (*C. sinensis*) dataset. **A:** Comparison of the number of highly expressed genes between the two haplotypes of each homologous chromosome. Data are shown as means ± s.d. **B:** Classification of ASE profiles across samples from various tissues and treatments, identifying five ASE groups. No_expression: Neither allelic gene pair is expressed; Diff00: no significant expression difference between a pair of alleles with *p*-adjust > 0.05; Diff0: significant difference between a pair of alleles with *p*-adjust ≤ 0.05 and |fold-change (FC)| ≤ 2; Diff2: significant difference between a pair of alleles with *p*-adjust ≤ 0.05 and 2 < |FC| < 8; Diff8: significant difference between a pair of alleles with *p*-adjust ≤ 0.05 and |FC| ≥ 8. **C–E:** Boxplots displaying *Ka* (number of substitutions per non-synonymous site), *Ks* (number of substitutions per synonymous site), and the *Ka*/*Ks* ratio for each allele pair in the three differentially expressed categories (Diff0, Diff2, and Diff8). The midline shows the 50th percentile and whiskers represent the minimum and maximum values. **F:** Absolute gene expression difference (in TPM) for the three differentially expressed categories of allele-specific gene expression.


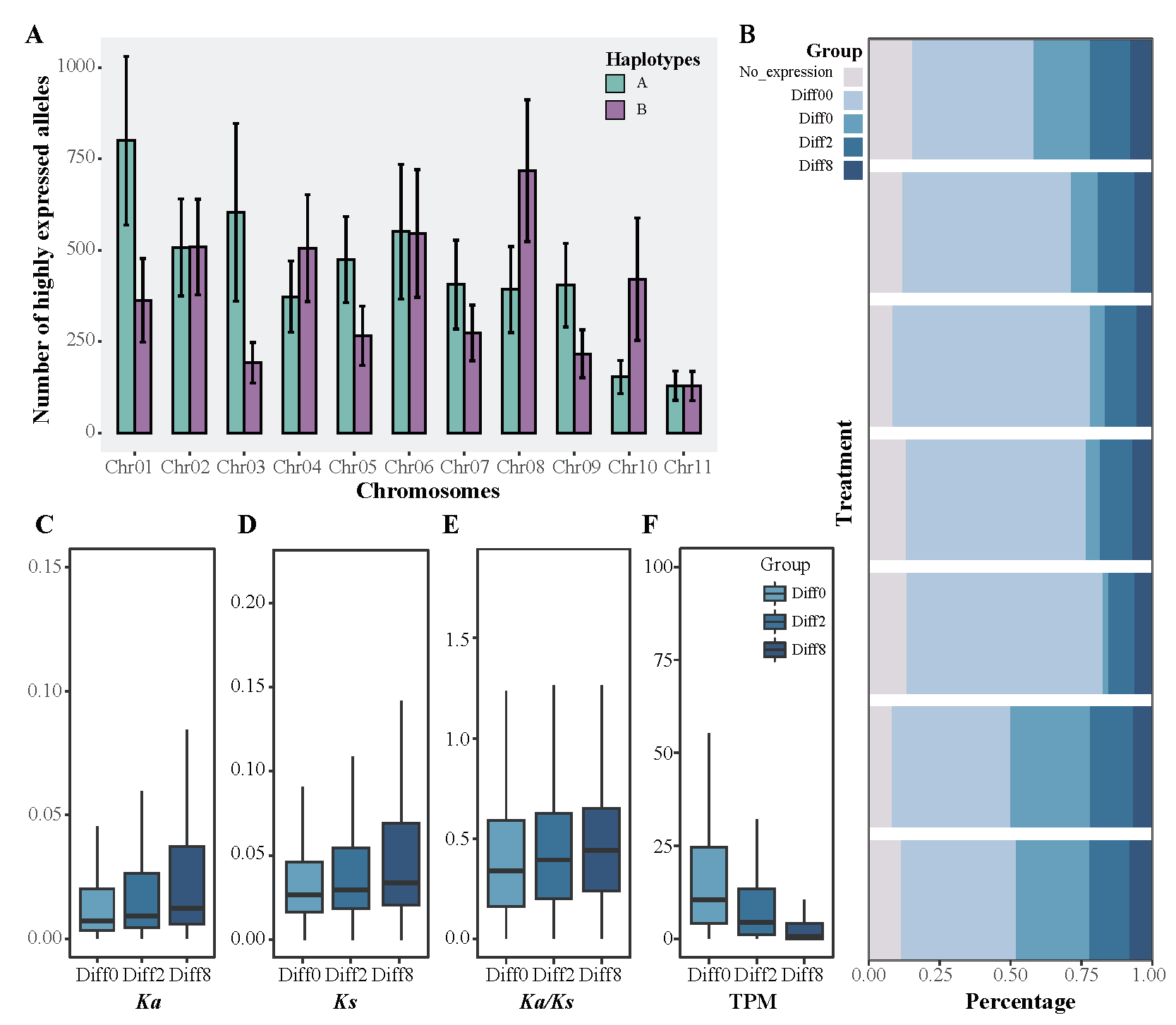


**Fig. S13.** ASE in the ginger (*Z. officinale*) dataset. **A:** Number of highly expressed genes in homologous chromosomes from different haplotypes. Data are shown as means ± s.d. **B:** Classification of ASE profiles across samples from various tissues and treatments, identifying five ASE groups. No_expression: Neither allelic gene pair is expressed; Diff00: no significant difference between a pair of alleles with *p*-adjust > 0.05; Diff0: significant difference between a pair of alleles with *p*-adjust ≤ 0.05 and |FC| ≤ 2; Diff2: significant difference between a pair of alleles with *p*-adjust ≤ 0.05 and 2 < |FC| < 8; Diff8: significant difference between a pair of alleles with *p*-adjust ≤ 0.05 and |FC| ≥ 8. **C–E:** Boxplots displaying *Ka*, *Ks*, and the *Ka*/*Ks* ratios for each allele pair in the three differentially expressed categories (Diff0, Diff2, and Diff8). The midline shows the 50th percentile and whiskers represent the minimum and maximum values. **F:** Absolute gene expression difference (in TPM) for the three differentially expressed categories of allele-specific gene expression.
