# Supplementary material for: Alleleauto: a pipeline for allele identification and analysis of allele-specific gene expression with haplotype-resolved diploid genome assemblies
Source: aBIOTECH. 2026 May 19;7(3):100056. doi: 10.1016/j.abiote.2026.100056 (PMC13240741; doi:10.1016/j.abiote.2026.100056)
Supplement: Multimedia component 3 [file mmc3.pdf]

Alleleauto

A pipeline for allele identification and analysis of allele-specific gene expression with haplotype-resolved diploid genome assemblies.

Version: 1.0.0

Author: Tian-Le Shi

Email: shitianle@baafs.net.cn

Link: https://github.com/shitianle77/Alleleauto

1 What is Alleleauto

Program: a pipeline for allele identification and analysis of allele-specific gene expression with haplotype-resolved diploid genome assemblies

Main commands:

[pipeline]

allele_identification for allele identification

allele_specific_expression for allele-specific expression analysis

For details on usage, please initiate the following commands:

```
bash allele_identification.sh -h
bash allele_specific_expression.sh -h
```

[Rule for allele-pair identification]

(1) The 3σ rule is used for allele-pair identification, please refer to its original publication for details (Lehmann, 2013).

(2) Tukey's method is an acclaimed and straightforward graphical technique known forrepresenting continuous univariate data through a boxplot. This method calculates the upper and lower extremes of the data through its quartiles.

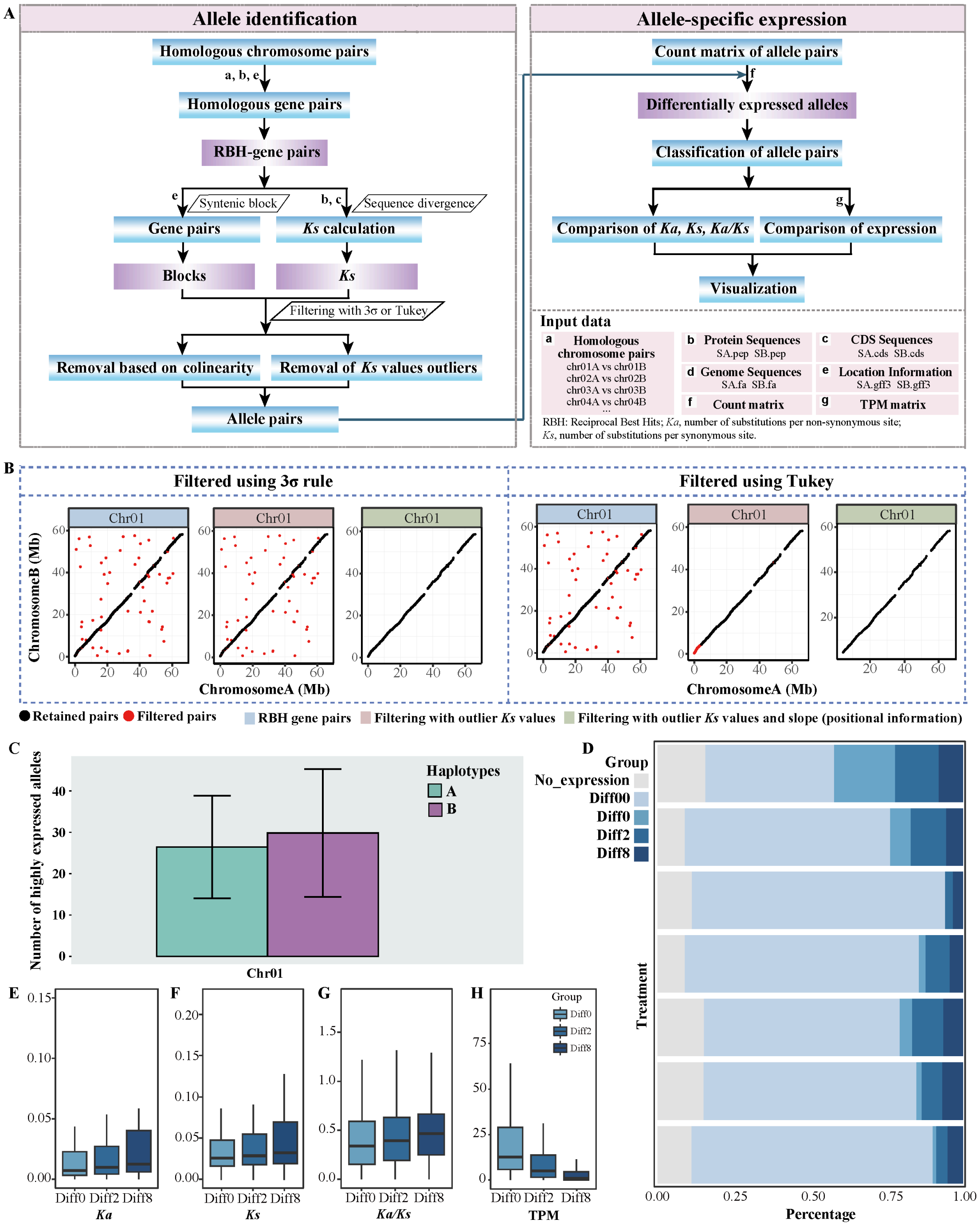

Fig. 1. Workflow and example output of the Alleleauto pipeline. **A.** Workflow of the Alleleauto pipeline. **B.** Identification and visualization of alleles (with filtering following the 3σ rule and Tukey's method). **C.** Number of highly expressed genes from different homologous chromosomes in different haplotypes. Data are shown as means ± standard deviation (s.d.). **D.** Grouping of allele-specific expression (ASE) profiles among samples from different tissues and treatments. No_expression: Neither allele is expressed; Diff00: the pair of alleles are not significantly differentially expressed with p-adjust > 0.05; Diff0: significant difference between a pair of alleles with p-adjust ≤ 0.05 and |fold-change (FC)| ≤ 2; Diff2: significant difference between a pair of alleles with p-adjust ≤ 0.05 and 2 < |FC| < 8; Diff8: significant difference between a pair of alleles with p-adjust ≤ 0.05 and |FC| ≥ 8. **E–G.** Boxplot visualization of *Ka*, *Ks*, and the *Ka*/*Ks* ratio for each allele pair from the three differentially expressed categories (Diff0, Diff2, and Diff8). The centerline represents the 50th percentile. The whiskers indicate the minimum and maximum values. **H.** Absolute difference in gene expression (in TPM) for the three differentially expressed categories of allele-specific gene expression.

2 Installation

2.1 Dependencies

Genetribe: https://github.com/chenym1/genetribe

WGDI: https://github.com/SunPengChuan/wgdi

2.2 Installation

2.2.1. Download the latest version of Alleleauto from Github We only provide a Linux 64-bit version.

```
git clone https://github.com/shitianle77/Alleleauto.git
```

2.2.2. Run the script to install Alleleauto

```
cd Alleleauto
conda env create -f environment.yml
conda activate Alleleauto

./install.sh
export PATH=/path/to/Alleleauto/genetribe:$PATH
```

2.2.3. Get helps on Alleleauto

```
cd Alleleauto
bash ./bin/allele_identification.sh -h
bash ./bin/allele_specific_expression.sh -h
```

3 Quick Start

We provide example files in the Alleleauto folder. Here we show steps on how to run a simple computation with this pipeline. We expect this may help users quickly become familiar with this pipeline.

The software is divided into two main sections, allele identification and allele-specific gene expression. All the input data are under the directory **00_data/**. You can keep only the directories **00_data/** and **bin/**, and then execute the following lines to obtain the results for the example data.

```
cd alleleauto
ls
# 00_data bin

# Allele identification (Filter using the 3σ rule)
bash ./bin/allele_identification.sh -p Zo_chrpairs.txt -a Zo_SA -b Zo_SB

# Filtering of allele pairs (Filter using Tukey's method)
bash ./bin/allele_filtering.sh -p Zo_chrpairs.txt -i 9

# Analysis of allele-specific expression
bash ./bin/allele_specific_expression.sh -a Zo_SA -b Zo_SB -c allelepairs.count_selected.
txt-t allelepairs.tpm_selected.txt -s 21
```

4 Input and output

Here we describe in detail the formats of the input files and the output files.

4.1 Files for the procedure of allele identification

- ### Input files

  (1) One such input file is for the grouping of homologous chromosomes.

  Produce an example of such input file by executing this command:

```
cat chrpairs.txt
# chr01A    chr01B  chr01
```

(2) Other input files include protein sequences (for example: SA.pep, SB.pep), coding sequences (SA.cds, SB.cds), sequences in fasta format (SA.fa, SB.fa), and gff files (SA.gff3, SB.gff3) from gene annotation and genome assembly of the two haploid (two haplotype-resolved genome assemblies) genomes.

Produce examples of such input files by executing the following commands:

```
cat SA.fa
# >chr01A
# taGCAAGTTGTTTTACCTAATTTATTTTAATGTTAAATATTTAGTATTTGTTGATAAAAAATATAAATCATAA…
cat SB.fa
# >chr01B
# ccaaatagttgatactacttgcccatgggtttcaaaggtatttgtttcccttttctaTCAGAGTAGAGAATAAGGTC…

cat SA.pep
# >Zioff01G0000100
# MNNASPSAAEPNSHALALPNPSSPLKDRSTYTNLKEHLLRPAGNNLWSPPVSKRATAGSKDVTRYRGVRRRPWGRYAA…
cat SB.pep
# >Zioff01G0466400
# MITTRFFPHSRFFLPSHLPTLCRPIHSGAAHPRITRSELVDRICRILTLERFHAIPKLPFRFSDDLLDAVLVRLRLD…

cat SA.cds
```

```
# >Zioff01G0000100
# ATGAATAATGCAAGTCCATCTGCTGCAGAACCCAACTCACACGCACTTGCTCTTCCTAATCCTTCTTCCCCACTTAA...
cat SB.cds
# >Zioff01G0466400
# ATGATCACAACTAGATTCTTCCCTCATTCGCGTTTCTTCCTCCCCTCGCACCTGCCCACTCTCTGCCGGCCCATCCA...

cat SA.gff3
# chr01A  maker  gene  1954  4056  .  + .  ID=Zioff01G0000100;Name=Zioff01G0000100
# chr01A  maker  mRNA  1954  4056  .  + .  ID=Zioff01G0000100.1;Parent=Zioff01G0000100
# chr01A  maker  exon  1954  2105  .  + .
ID=Zioff01G0000100.1:exon:1170;Parent=Zioff01G0000100.1
# chr01A  maker  exon  3204  4056  .  + .
ID=Zioff01G0000100.1:exon:1171;Parent=Zioff01G0000100.1
# chr01A  maker  CDS  1954  2105  .  +  0
ID=Zioff01G0000100.1:cds;Parent=Zioff01G0000100.1
cat SB.gff3
# chr01B  maker  gene  2566  8399  .  + .  ID=Zioff01G0466400;Name=Zioff01G0466400
# chr01B  maker  mRNA  2566  8399  .  + .  ID=Zioff01G0466400.1;Parent=Zioff01G0466400
# chr01B  maker  exon  2566  2954  .  + .
ID=Zioff01G0466400.1:exon:1;Parent=Zioff01G0466400.1
# chr01B  maker  CDS  2680  2954  .  +  0
ID=Zioff01G0466400.1:cds;Parent=Zioff01G0466400.1
```

- ## Output files

The expected output files are written in different folders. Here, we have “raw_RBH.genepairs”, “RBH.genepairs”**, and** “SA_SB.blast” **in the** “01_genetribe” **folder, and we have files of** “SA_SB.collinearity.txt”, “SA_SB.ks.txt”**,** “SA_SB_block.csv”**,** “block.tsv”**,** “filtered.block.tsv”**,** “genepairs_info.tsv”**,** “filtered.genepairs_info.tsv”**, and** “allele_pairs_lasted.txt” **in the** “02_wgdi” **folder.**

Get into different folders for details, by executing the following commands:

```
cd 01_genetribe
```

Output files	Description
raw_RBH.genepairs	Gene pairs belonging to the Reciprocal Best Hits (RBH)
RBH.genepairs	List of gene pairs belonging to RBH
SA_SB.blast	Blast information for gene pairs belonging to RBH

For more information, please refer to: https://chenym1.github.io/genetribe/tutorial/fileformats.html

```
cd 02_wgdi
```

Output files	Description
SA_SB.collinearity.txt	Improved collinearity (for details, see https://wgdi.readthedocs.io/en/latest/collinearity.html)

Output files	Description
SA_SB.ks.txt	Non-synonymous (Ka) and synonymous (Ks) (for details, see https://wgdi.readthedocs.io/en/latest/ks.html)
SA_SB_block.csv	BlockInfo (for details, see https://wgdi.readthedocs.io/en/latest/blockinfo.html)
block.tsv	BlockInfo (same as SA_SB_block.csv, separated by tabs)
filtered.block.tsv	Filtered blocks information
genepairs_info.tsv	Details of allele pairs on blocks (position and Ks information)
filtered.genepairs_info.tsv	Details of filtered allele pairs on blocks (position and Ks information)
allele_pairs_lasted.txt	The final allele pairs identified

```
cd 02_wgdi-Tukey
```

Output files	Description
allele_pairs_lasted.txt	The final allele pairs identified
block.tsv	BlockInfo (same as SA_SB_block.csv, separated by tabs)
genepairs_info.tsv	Details of allele pairs on blocks (position and Ks information)
filtered_ks_NG86-1.5.genepairs_info.tsv	Details of filtered allele pairs after removing Ks outliers (position and Ks information)
filtered_slope-1.5.genepairs_info.tsv	Details of filtered allele pairs after removing slope outliers (position and Ks information)
filtered.ks-slope-bioplot_genepairs_info.tsv	Details of filtered allele pairs after removing Ks and slope outliers (position and Ks information)

```
cd 02_wgdi-Tukey/allele_plot
```

Output files	Description
chr01.coord.allele	Details of the position information of the filtered allele pairs on chromosome 1
pairs.coord.allele	Details of the position information of the filtered allele pairs
pairs.allele.pdf	Dotplot plot of filtered allele pairs

4.2 Files for computation of allele-specific expression

- ## Input files

The two core input files are of the count and TPM expression matrices for the allele pairs.

The count matrix has the following format; and the TPM matrix has the same format.

Allele_ID	SA_s1_1	SA_s1_2	SA_s1_3	SA_s2_1	SA_s2_2	SA_s2_3	...	SB_s1_1	SB_s1_2	SB_s1_3	SB_s2_1	SB_s2_2	SB_s2_3	...
allele1A_allele1B	0	0	0	0	0	0	...	0	0	0	0	0	0	...
allele2A_allele2B	0	0	0	0	0	0	...	0	0	0	0	0	0	...
allele3A_allele3B	780.324	906.261	796.327	0	0	0	...	712.676	832.739	733.673	0	0	0	...
...														

SA_s1_1: The first repeat (1) of the allele of haplotype A (HA) in sample 1 (s1).

SB_s2_3: The third repeat (3) of the allele of haplotype B (HB) in sample 2 (s2).

Here is one example (The count values of allele pairs in the three replicates of the first sample.):

```
cd ./00_data/RNA_seq
cat allelepairs.count_selected.txt
# Zioff01G0000100-Zioff01G0466400   0   0   0   0   0   0
# Zioff01G0000200-Zioff01G0466500   828 907 757 354 384 406
# Zioff01G0000300-Zioff01G0466600   0   0   0   0   0   0
# Zioff01G0000400-Zioff01G0466700   2619    2758    2141    3305    4140    3122
# Zioff01G0000500-Zioff01G0466800   15  21  10  0   0   0
```

- ## Output files

  The expected output files are written in different folders. Here, we have “pairs.genelist”**,** “name_list.txt”**,** “AvsB.”**,** “stats_number/”**, and** “High_expression/” **in the** “03_DEG/1_Class_alleles” **folder, and we have** “.name”**,** “.tpm”**,** “tpm.box.txt”**,** “tpm_boxplot.pdf” **in the** “03_DEG/2_Diff_comparison” **folder, and also** “all.kaks” **and** “.pdf”**, in the “KaKs” folder.**

```
cd 03_DEG/1_Class_alleles
```

Output files	Description
pairs.genelist	Allele pairs
name_list.txt	Name of samples
AvsB.	Differentially expressed alleles in all samples of haplotypes A and B, respectively (“up” represents alleles that are highly expressed in haplotype A compared to haplotype B)
stats_number/	Multiple statistics of allele expression in different tissues or treatments
High_expression/	The number of highly expressed alleles in each chromosome

```
cd 03_DEG/2_Diff_comparison
cd TPM
```

Output files	Description
.name	The pairs of differentially expressed alleles in Diff0, Diff2 and Diff8
.tpm	TPM values of differentially expressed allele pairs in Diff0, Diff2 and Diff8
tpm.box.txt	Summary of tpm values for the differentially expressed alleles in each group (Diff0, Diff2 and Diff8)
tpm_boxplot.pdf	Distribution diagram of alleles with different differential expression folds under each group (Diff0, Diff2 and Diff8)

```
cd KaKs
```

Output files	Description
all.kaks	Summary of Ka, Ks and Ka/Ks values between allele pairs in each group (Diff0, Diff2 and Diff8)
.pdf	Distribution diagram of Ka, Ks and Ka/Ks between allele pairs in each group (Diff0, Diff2 and Diff8)

References to different file formats in the above bioinformatics pipeline:

fasta: https://en.wikipedia.org/wiki/FASTA_format

gff: https://en.wikipedia.org/wiki/General_feature_format

bed: https://en.wikipedia.org/wiki/BED_(file_format)

5 Parameter setting

5.1 Parameter setting in the allele identification step

5.1.1 This can be executed by initiating a single-line command. One exemplar command is:

```
bash ./bin/allele_identification.sh -p chrpairs.txt -a SA -b SB
```

Parameter	Description
-p	Necessary parameter. the target chromosome list
-a	Necessary parameter. name of haplotype A

Parameter	Description
-b	Necessary parameter. name of haplotype B
-h	Print brief help message

Note: The names of the haplotypes and the prefixes of the input files must be the same (e.g.: If -a is SA, the input file will be SA.pep; if -a is HaplotypeA, the input file will be HaplotypeA.pep.). The parameter setting requirements in 5.2 are the same.

5.1.2 This can be executed by initiating a single-line command. One exemplar command is:

```
bash ./bin/ allele_fiiltering.sh -p chrpairs.txt -i 9
```

Parameter	Description
-p	Necessary parameter. The target chromosome list.
-i	Necessary parameter. The number of Inter Quartile Range (IQR).

Tukey IQR Multiplier (-i) Parameter Guide

(1) The -i parameter in Alleleauto filter controls Tukey's Interquartile Range (IQR) method for removing outlier allele pairs based on their synonymous substitution rate (Ks). A pair is flagged as an outlier if its Ks falls outside: **[Q1 − i × IQR, Q3 + i × IQR]**, where Q1 and Q3 are the 25th and 75th percentiles of the Ks distribution, and IQR = Q3 − Q1.

Smaller i = narrower acceptance window = more pairs removed (stricter).

Larger i = wider acceptance window = fewer pairs removed (more permissive).

(2) Tukey filtering is optional. It is useful when:

- The Step 1 allele table contains residual noise from ancient whole-genome duplication (WGD) events or translocated segments.
- The colinearity dot plot shows off-diagonal blocks that are not true allelic relationships.
- You want to refine allele pairs for downstream expression analysis as false positives would bias ASE classification.

  If Step 1 already produces a clean dot plot with pairs concentrated along the diagonal, Tukey filtering may not be necessary.

5.2 Parameter setting in the allele-specific expression (ASE) step

This can be executed by initiating a single-line command. One exemplar command is:

```
bash ./bin/allele_specific_expression.sh -a SA -b SB -c allelepairs.count_selected.txt -t
allelepairs.tpm_selected.txt -s 21
```

Parameter	Description
-a	Necessary parameter. Name of haplotype A.
-b	Necessary parameter. Name of haplotype B.
-c	Necessary parameter. Count matrix of allele pairs.
-t	Necessary parameter. Tpm matrix of allele pairs.
-s	Necessary parameter. Number of samples.
-h	Print brief help message.

6 Note

In the subcommands tpm_boxplot.r (line 27) and kaks_boxplot.r (lines 28, 43 and 58) of allele_specific_expression.sh, users can adjust according to their actual results if necessary.
